# Control of Clinical Pathogens by the Haemolymph of *Paratelphusa hydrodromous*, a Freshwater Crab

**DOI:** 10.5402/2011/642768

**Published:** 2011-05-19

**Authors:** A. Arul Prakash, S. Balasubramanian, G. Gunasekaran, M. Prakash, P. Senthil Raja

**Affiliations:** Department of Zoology, Annamalai University, Annamalainagar, Tamilnadu 608 002, India

## Abstract

In the present study, effort has been made to find the antimicrobial activity of haemolymph collected from freshwater crab, *Paratelphusa hydrodromous*. The haemolymph collected was tested for antimicrobial assay by disc diffusion method against clinical pathogens. Five bacterial species, namely, *Escherichia coli, Klebsiella pneumonia, Proteus mirabilis, Pseudomonas aeruginosa, Staphylococcus aureus,* and five fungal strains, namely and *Aspergillus flavus, Aspergillus fumigatus, Aspergillus niger, Rhizopus sp., and Mucor sp.,* were selected for the study. The result shows a strong response of haemolymph against the clinical pathogens which confirms the immune mechanism of the freshwater crab.

## 1. Introduction

Antimicrobial peptides are a major component of the innate immune defense system in invertebrates [1]. Invertebrates represent the most diverse taxon of animals on the planet, accounting for more species than all other animals combined. In addition to that experimental evidence, it makes sense that such successful animals would have evolved efficient means for combating infection. In many ways, invertebrates face the similar immune challenges to those experienced by vertebrates [2]. As a consequence, invertebrates have to rely on the innate immune processes to combat pathogens which closely resemble the innate immune system of the vertebrates.

The invertebrates comprise over 95% of the animal species, and some live in environments rich in potentially harmful microorganisms. As a result, these animals have developed various competent strategies to defend their lives against invading pathogens [3]. Living in an aquatic environment rich in microorganisms, crustaceans have developed effective system for detecting and eliminating noxious microorganisms. The crabs are in intimate contact with aquatic environment rich in pathogenic microbes and are prone to infection by those microbes at various stages of growth, and losses due to disease can be enormous [4].

Antimicrobial peptides or substances are the host defense compounds that have recently drawn attention, due to their properties and diversity. In crustaceans, these have substances are considered to be a main component of innate immunity [5]. There are few reports evaluating the bioactivity of crustaceans, and many researchers have studied the antibacterial activity of marine crustaceans and prawns [6–10]. The crabs are the rich sources of bioactive compounds, but the researchers carried out the pharmacological properties of marine crabs [11, 12] but not in freshwater crabs. Hence, the present study was aimed to investigate the antimicrobial potency of haemolymph collected from freshwater crab, *Paratelphusa hydrodromous*.

## 2. Materials and Methods

### 2.1. Collection of Animals

The healthy crabs, *Paratelphusa hydrodromous, *were collected from different sites of paddy fields in Pethankuppam village, Cuddalore District, Tamilnadu, India and brought to the laboratory for the collection of haemolymph.

### 2.2. Collection of Haemolymph

Healthy crabs at different stages of development were used throughout the experimental period, and crabs are subjected to a single bleed collection. Haemolymph (approx. 3 mL) was collected by cutting the leg live animal with fine sterile scissors.

To avoid haemocytes degranulation and coagulation, the haemolymph was collected in the presence of sodium citrate buffer pH 4.6 (2.1 v/v). Equal volume of physiological saline (0.85% Nacl, w/v) was also added to it. To remove the haemocytes from haemolymph, the haemolymph was centrifuged at 2000 g for 15 min at 4°C. Supernatant was collected and stored at 4°C until use.

### 2.3. Microbial Strains Used

Antimicrobial activity of freshwater crab haemolymph was determined against 5 bacterial strains, namely, *Escherichia coli, Klebsiella pneumonia, Proteus mirabilis, Pseudomonas aeruginosa, and Staphylococcus aureus,* and 5 fungal strains, namely, *Aspergillus flavus, Aspergillus fumigatus, Aspergillus niger, Rhizopus sp., and Mucor sp.*

These pathogenic strains were obtained from the Division of Microbiology, Rajah Muthiah Medical College, Annamalai University, Annamalai Nagar.

### 2.4. Antimicrobial Assay

The spectrum of antimicrobial activity was studied using the above-mentioned bacteria and fungi, which are designated as human pathogens. erythromycin was used as a positive control for bacteria and fluconazole for pathogenic fungi. 

*In vitro* antibacterial assay was carried out by the disc diffusion technique [13]. Whatman No.  1 filter paper discs with 4 mm diameter were impregnated with known 10 *μ*L of test sample (crab haemolymph), and 10 *μ*g/mL positive control contained a standard antibiotic disc. Negative controls (sterile disc only) are also used. The impregnated discs along with control were kept on nutrient agar plates, seeded with test bacterial cultures and potato dextrose agar fungal culture separately. At room temperature (37°C), the bacterial plates were incubated for 24 hrs. The fungal plates were incubated at 30°C for 48 hrs to find out the antimicrobial activity. They were expressed in terms of diameter of zone of inhibition, measured in mm using cm scale, and recorded. In each strain, 5 replicates were maintained, and mean was tabulated along with their standard error. *t*-test values were found and incorporated in the table. The * mark represents the significant difference at 5% level. The haemolymph of crab had more zone of inhibition in *E. coli* and *Rhizopus sp*. So only for these two the significance was calculated by using *t*-test and was tabulated. For all the others, the *t*-test values were not calculated.

## 3. Results

The antimicrobial effect of haemolymph collected from freshwater crab, *Paratelphusa hydrodromous,* was tested against five human pathogenic bacteria, namely, *Escherichia coli*, *Klebsiella pneumonia*, *Pseudomonas aeruginosa*, *Proteus mirabilis*, and *Staphylococcus aureus* and five pathogenic fungal species, namely, *Aspergillus flavus*, *Aspergillus niger*, *Aspergillus fumigatus*, *Rhizopus,* and *Mucor*.

### 3.1. Antibacterial Effect of Haemolymph from *Paratelphusa hydrodromous*

The screening of the haemolymph from *Paratelphusa hydrodromous* showed a significant bactericidal activity with regard to the Gram-positive as well as Gram-negative bacteria. The zone of inhibition values of haemolymph of *Paratelphusa hydrodromous* are compared with a positive control and negative control. The values are presented in Table 1.

The haemolymph of *Paratelphusa hydrodromous* showed a significant or more effect in controlling the growth of Gram-negative bacteria, *Escherichia coli,* with an inhibition zone of 17 mm in diameter which is more than the positive control. 

Next to *Escherichia coli*, the haemolymph of *Paratelphusa hydrodromous* showed a better effect on *Klebsiella pneumonia* having an inhibition zone of 16 mm in diameter. That was followed by the *Pseudomonas aeruginosa* with an inhibition zone of 15 mm in diameter. Among the four Gram-negative bacteria tested, *Proteus mirabilis* showed very less sensitivity to the haemolymph of *Paratelphusa hydrodromous* with an inhibition zone of 12 mm in diameter. The haemolymph of *Paratelphusa hydrodromous* showed the effect on the Gram-positive bacteria *Staphylococcus aureus*, with an inhibition zone of 13 mm in diameter.

The antibacterial activity of positive control erythromycin showed a maximum activity against *Klebsiella pneumonia *(26 mm), *Pseudomonas aeruginosa *(25 mm), *Proteus mirabilis* (23 mm), *Staphylococcus aureus* (20 mm), and *Escherichia coli *(15 mm), respectively.

### 3.2. Antifungal Effect of Haemolymph from *Paratelphusa hydrodromous*

The effect of haemolymph from *Paratelphusa hydrodromous* against five pathogenic fungi, *Aspergillus flavus, Aspergillus niger, Aspergillus fumigatus, Rhizopus, and Mucor,* showed variation in its zone of inhibition. The zone of inhibition values of haemolymph of *Paratelphusa hydrodromous* were compared with a positive control, and the values are presented in Table 2. The haemolymph of *Paratelphusa hydrodromous* showed more effect in controlling the growth of *Aspergillus flavus* with an inhibition zone of 22 mm in diameter, which shows the highest zone of inhibition among tested fungi, but the zone of inhibition is less than the positive control.

Next to the *Aspergillus flavus,* the haemolymph of *Paratelphusa hydrodromous* showed better effect on *Rhizopus *having an inhibition zone of 18 mm. The zone of inhibition in *Rhizopus *by the haemolymph is significantly higher than the positive control.

The antifungal activity of *Paratelphusa hydrodromous* haemolymph against *Aspergillus niger* has the zone of inhibition 13 mm. Haemolymph of *Paratelphusa hydrodromous* was noted to have 13 mm zone of inhibition against *Mucor*, whereas *Aspergillus fumigatus* was observed with an inhibition zone of 12 mm.

The antifungal activity of positive control fluconazole showed a maximum activity against *Aspergillus flavus *(25 mm), and this activity was followed by *Aspergillus niger* (21 mm), *Aspergillus fumigatus *(19 mm), *Mucor *(19 mm), and *Rhizopus *(16 mm), respectively.

## 4. Discussion

Invertebrates lack an adaptive immune system. The recognition of the pathogens and parasites by the invertebrate immune system may involve soluble proteins present in the haemolymph as well as proteins localized at the surface of the haemocytes or other cells [13]. Antibacterial peptides can also be induced in epidermal cells in response to wounding or infection in the cuticles [14]. The whole process of synthesizing antibacterial proteins may take few minutes or hours after the changes, and these are secreted into the haemolymph of which some are lysozyme [15] and andropin [16]. These proteins show strong resistance to the microbial growth. The result of the present work is in agreement with the above reports, where the haemolymph collected from the freshwater crab, *Paratelphusa hydrodromous,* exerts strong activity against the tested microbes. 

It has been observed that, in various invertebrate species, bacteria injected into the haemocoel elicit the synthesis of a number of antimicrobial peptides and proteins, which are recreated into the haemolymph and are active [2]. As the haemolymph showed antibacterial activity, it offers to suggest that broad spectrum of antibacterial peptides was secreted in response to immunization [14].

In arthropods, antimicrobial compounds were mainly studied in chelicerates (crabs and insects). The involvement of microbial activity is quite different in crabs and insects. In crabs, the antimicrobial compounds are synthesized in haemocytes, where they are stored after processing within their cytoplasmic granules [17]. Their substances are released into haemolymph through regulated exocytosis upon microbial stimulation. The presence of antimicrobial compounds in the haemolymph of crustacean species (crabs) has been reported by so many researchers [9, 11, 18].

Following these in our present study, the crab haemolymph showed strong activity against the growth of selected microbes. The result suggests that the crab can produce antimicrobial substances instantly to combat microbial infection. In crustaceans, antibacterial peptides have been isolated form the granular haemocytes of shore crabs (*Carcinius maenas*) [19] and the haemolymph of penaeid shrimp [20]. 

The defence system of crustacean against microbes rests largely on cellular activities performed by haemocytes/haemolymph such as adhesion, phagocytosis, encapsulation, and melanisation. Crabs haemolymph is known to contain several immune effects, and they play a major role in the innate immune mechanisms. In conclusion, this study shows that the haemolymph of freshwater crab, *Paratelphusa hydrodromous,* may contain several substances with antimicrobial activity. The revealing and development of the antimicrobial compounds in the haemolymph will provide an opportunity for the production of new compounds with natural activities as an alternative to antibiotics. 

Further purification of the active compounds is necessary in order to identify their chemical nature and to evaluate their potency as a novel drug.

## Figures and Tables

**Table 1:** Antibacterial activity of haemolymph from *Paratelphusa hydrodromous. *

Sl. no.	Pathogens	Zone of inhibition in mm			−ve control mm
		+ve control	Test sample	*t*-test value	
(1)	*Escherichia coli*	14.83 ± 0.43	17.16 ± 0.85	2.47*	0
(2)	*Klebsiella pneumonia*	25.73 ± 0.42	16.05 ± 0.35	—	0
(3)	*Pseudomonas aeruginosa*	24.87 ± 0.10	15.15 ± 0.51	—	0
(4)	*Proteus mirabilis*	23.2 ± 0.71	12.18 ± 0.50	—	0
(5)	*Staphylococcus aureus*	20.48 ± 0.75	12.78 ± 0.65	—	0

*Significant at 5% level.

**Table 2:** Antifungal activity of haemolymph from *Paratelphusa hydrodromous. *

Sl. no.	Pathogens	Zone of inhibition in mm			−ve control mm
		+ve control	Test sample	*t*-test value	
(1)	*Aspergillus flavus*	24.9 ± 0.59	21.66 ± 0.57	—	0
(2)	*Aspergillus niger*	21.25 ± 0.82	13.37 ± 0.68	—	0
(3)	*Aspergillus fumigatus*	18.83 ± 0.86	12.08 ± 0.53	—	0
(4)	*Rhizopus sp.*	15.73 ± 0.80	18.45 ± 0.50	2.89*	0
(5)	*Mucor sp.*	19.43 ± 0.69	13.16 ± 0.54	—	0

*Significant at 5% level.

## References

[1]  Tincu JA, Taylor SW (2004). Antimicrobial peptides from marine invertebrates. *Antimicrobial Agents and Chemotherapy*.

[2] Gillespie JP, Kanost MR, Trenczek T (1997). Biological mediators of insect immunity. *Annual Review of Entomology*.

[3] Jiravanichpaisal P, Lee BL, Soderhall K (2006). Cell-mediated immunity in arthropods: hematopoiesis, coagulation, melanization and opsonization. *Immunobiology*.

[4] Hudson DA, Lester RJG (1994). Parasites and symbionts of wild mud crabs *Scylla serrata* (Forskal) of potential significance in aquaculture. *Aquaculture*.

[5] Smith VJ, Chisholm JRS (2001). Antimicrobial proteins in crustaceans. *Advances in Experimental Medicine and Biology*.

[6] Tonganunt M, Wongmanee K, Sartthai S, Chotigeat W, Phongdara A (2008). Crustin protein Amk1 from black tiger shrimp (*Penaeus monodon*) inhibits *Vibrio harveyi* and *Staphylococcus aureus*. *Songklanakarin Journal of Science and Technology*.

[7] Stewart JE, Zwicker BM (1972). Natural and induced bactericidal activities in the haemolymph of the lobster *Homarus americanus*: products of haemocyte-plasma interactions. *Canadian Journal of Microbiology*.

[8] Noga EJ, Arroll TA, Fan Z (1996). Specificity and some physicochemical characteristics of the antibacterial activity from blue crab *Callinectes sapidus*. *Fish and Shellfish Immunology*.

[9] Khoo L, Robinette DW, Noga EJ (1999). Callinectin, an antibacterial peptide from blue crab, *Callinectes sapidus*, hemocytes. *Marine Biotechnology*.

[10] Ravichandran S, Wahidulla S, Souza LD, Rameshkumar G (2009). Antimicrobial lipids from the hemolymph of brachyuran crabs. *Applied Biochemistry and Biotechnology*.

[11] Veeruraj A, Ravichandran S, Rameshkumar. G (2008). Antibacterial activity of crab haemolymph on clinical pathogens. *Trends in Applied Sciences Research*.

[12] Anbuchezien RM, Ravichandran S (2009). Influence of crab haemolymph on clinical pathogens. *Advanced Biology*.

[13] Bauer AW, Kirby WM, Sherris JC, Turck M (1966). Antibiotic susceptibility testing by a standardized single disk method. *American Journal of Clinical Pathology*.

[14] Hoq MI, Seraj MU, Chowdhury S (2003). Isolation and characterization of antimicrobial peptides form the mud crab, *Scylla serrata*. *Pakistan Journal of Biological Sciences*.

[15] Lee WJ, Brey PT (1995). Isolation and characterization of the lysozyme-encoding gene from the silkworm *Bombyx mori*. *Gene*.

[16] Samakovilis CP, Kylsten DA, Kimbrell A, Engstrom A, Hultmark D (1991). The adropoin gene and its product, a male specific antibacterial peptide in *Drosophilla melanogaster*. *The EMBO Journal*.

[17] Schnapp D, Kemp GD, Smith VJ (1996). Purification and characterization of a proline-rich antibacterial peptide, with sequence similarity to bactenecin-7, from the haemocytes of the shore crab, *Carcinus maenas*. *European Journal of Biochemistry*.

[18] Chisholm JRS, Smith VJ (1992). Antibacterial activity in the haemocytes of the shore crab, *Carcinus maenas*. *Journal of the Marine Biological Association of the United Kingdom*.

[19] Destoumieux D, Bulet P, Loew D, Van Dorsselaer A, Rodriguez J, Bachere E (1997). Penaeidins, a new family of antimicrobial peptides isolated from the shrimp *Penaeus vannamei* (*Decapoda*). *The Journal of Biological Chemistry*.

[20] Powning RF, Davidson WJ (1973). Studies on insect bacteriolytic enzymes. I. Lysozyme in haemolymph of *Galleria mellonella* and *Bombyx mori*. *Comparative Biochemistry and Physiology*.

